# First evidence of vertical *Hepatozoon canis* transmission in dogs in Europe

**DOI:** 10.1186/s13071-022-05392-7

**Published:** 2022-08-23

**Authors:** Ingo Schäfer, Elisabeth Müller, Ard M. Nijhof, Heike Aupperle-Lellbach, Gerhard Loesenbeck, Sybille Cramer, Torsten J. Naucke

**Affiliations:** 1 Laboklin GmbH and Co. KG., Bad Kissingen, Germany; 2 Institute for Parasitology and Tropical Veterinary Medicine, Freie Universität Berlin, Berlin, Germany; 3 Tierärztliche Praxis Jens Cramer, Ganderkesee, Germany

**Keywords:** Hepatozoonosis, Canine, Transplacental, Infection, *Hepatozoon canis*

## Abstract

**Background:**

*Hepatozoon canis* is a protozoal agent that is known to be transmitted by oral uptake of *H. canis*-infected *Rhipicephalus sanguineus* sensu lato ticks in dogs. Vertical transmission of *H. canis* has only been described once in a study evaluating dogs from Japan. The aim of this study was to investigate the parasitological status of puppies from a bitch that had tested positive for *Hepatozoon* spp. prior to giving birth.

**Findings:**

A 4-year-old, female, pregnant dog imported from Italy (Sardinia) to Germany showed clinical signs of lethargy and tachypnoea and tested positive for *H. canis* by PCR. The dog gave birth to eight puppies, one of which was stillborn and another that had to be reanimated. Haematology, buffy coat analysis and a biochemistry profile were performed for each dog. EDTA-blood of the surviving seven puppies and bone marrow, liver, spleen, amniotic fluid, and umbilical cord of the stillborn puppy was tested for the presence of *Hepatozoon* spp. by PCR.

The mother and the seven surviving puppies tested positive for *H. canis* by PCR at day 62 post-partum. Gamonts were detected in all dogs by buffy coat evaluation. Haematological and biochemistry results revealed mild abnormalities. In the stillborn puppy, spleen, umbilical cord, and amniotic fluid were positive for *H. canis*.

**Conclusion:**

The results confirm that vertical transmission is a possible route of *H. canis* infection in dogs, demonstrated by molecular detection of the pathogen in the stillborn puppy. In the seven surviving puppies, vertical transmission was the most likely transmission route. A potential impact of the level of parasitaemia on the health of puppies, as well as its pathogenesis, should be investigated further.

**Graphical Abstract:**

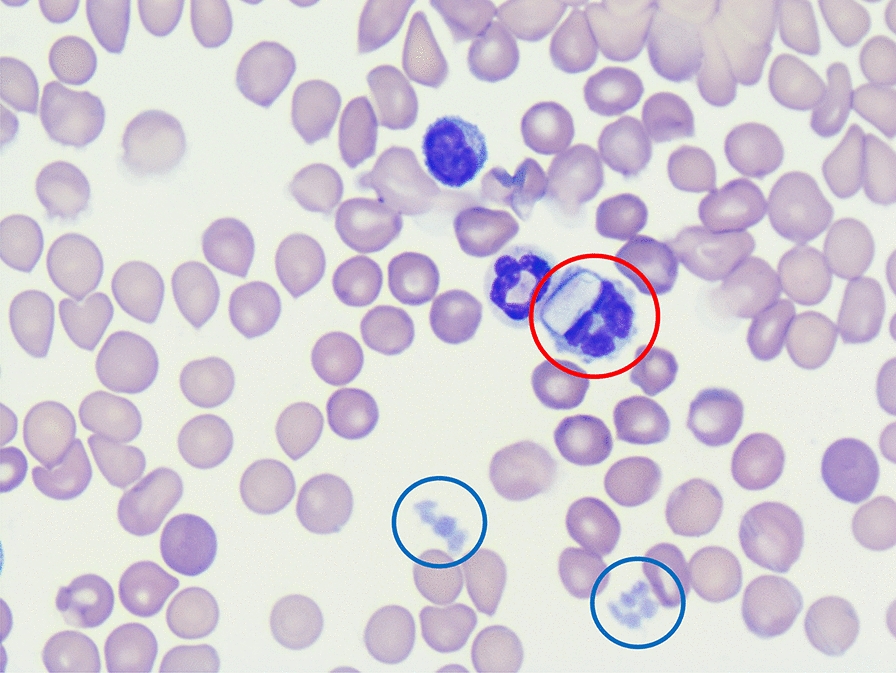

**Supplementary Information:**

The online version contains supplementary material available at 10.1186/s13071-022-05392-7.

## Main text

*Hepatozoon canis*, *H. americanum* and other *Hepatozoon* spp. are protozoal, apicomplexan agents. Blood-feeding arthropods are known as biological vectors for *Hepatozoon* spp., and their vertebrate hosts include dogs, cats, rodents, birds, reptiles and amphibians, which are susceptible to and affected by potential infection [1, 2]. In Germany, *H. canis* was previously detected by polymerase chain reaction (PCR) in 0–11% of dogs with a travel history [3–7]. In dogs in Europe, *H. canis* is thought to mainly be transmitted by the ingestion of *Rhipicephalus* *sanguineus* sensu lato (s.l.) ticks harbouring mature oocysts [8]. In northern Germany, a large proportion of the fox population tested positive for *H. canis* by PCR, with genotypes typically found in dogs [9]. Thus, foxes are thought to be potential pathogen reservoirs and alternative tick vectors, or alternative transmission routes are likely to be responsible for transmission within the fox population, as *R. sanguineus* s.l. ticks are not endemic in northern Germany [9]. *Hepatozoon canis* oocysts have also been detected in other tick species, including *Rhipicephalus microplus*, *Haemaphysalis longicornis* and *Haemaphysalis flava* [10, 11]. *Amblyomma ovale* was experimentally shown to be a suitable vector for *H. canis* in South America [12], but none of these tick species is endemic in Germany. *Ixodes ricinus* is widespread in Europe and *H. canis* DNA was detected in one *I. ricinus* tick collected from the environment in Italy [13], but additional studies suggested that this tick species does not act as a vector for *H. canis* [14]. Transstadial transmission of *H. canis* from *R. sanguineus* s.l. larvae to nymphs has been described [15].

The life-cycle for all *Hepatozoon* spp. includes gamogony and sporogony in haematophagous invertebrate definitive hosts and merogony and gametogony in vertebrate hosts [1]. After ingestion of a tick harbouring oocytes that contain sporozoites, the infective sporozoites are released in the gastrointestinal tract of the vertebrate host and reach blood and lymph circulation by penetrating the gut wall. Merogony starts in lymphoid tissues such as the bone marrow and from 13 days post-infection onwards, merozoites penetrate neutrophilic granulocytes and monocytes to develop into gamonts [16]. During the tick’s blood meal on an infected host, the gamonts are ingested and gametogenesis takes place in the gut of the tick, followed by sporogony in the haemocoel [1]. Besides vectorial transmission, additional transmission pathways of *Hepatozoon* spp. have been described and include predation of infected animals, although this has not been described for *H. canis* [1, 17]. Intrauterine transmission of *H. canis* was previously demonstrated in a study from Japan, in which *H. canis* gamonts were observed in peripheral blood smears in 23 out of 29 puppies (79%) from a total of six deliveries at 16 to 60 days after birth [18].

Dogs infected with *H. canis* usually do not show any clinical signs. Dogs may show clinical signs if a high level of parasitaemia is reached or if co-infections with other vector-borne infectious agents occur [19]. Clinical manifestation may be severe, e.g. lethargy, fever, anorexia, weight loss, lymphadenomegaly and anaemia (16, 17). In one study, 28 dogs younger than 18 months naturally infected with *H. canis* confirmed by PCR testing and cytology did not show any clinical signs [19], but haematological abnormalities were present in 26 out of the 28 dogs (93%); mainly eosinophilia (77%), leucocytosis (46%), lymphocytosis (31%), neutrophilia (23%), monocytosis (19%), thrombocytopenia (19%) and anaemia (4%). However, 13 out of these 26 dogs (50%) with haematological abnormalities were co-infected with other vector-borne pathogens [19].

The diagnosis of hepatozoonosis is most frequently based on PCR results [20, 21], as PCRs have higher sensitivity and specificity compared to other diagnostic tools such as microscopic evaluation [22]. Gamonts often are incidental findings when analysing blood smears. Additionally, histopathology may reveal meronts and/or monozoic cysts in different tissues [23]. Serological tests, such as the immunofluorescence antibody test (IFAT), detect antibodies against *H. canis* with high sensitivity mainly in dogs with chronic infections [24, 25], but are not used routinely.

To the best of the authors’ knowledge, vertical transmission of *H. canis* has not been reported from dogs in Europe until now. We therefore performed a follow-up on the history of a female pregnant bitch imported from Italy to Germany which previously tested positive for *H. canis*.

**Fig. 1:**
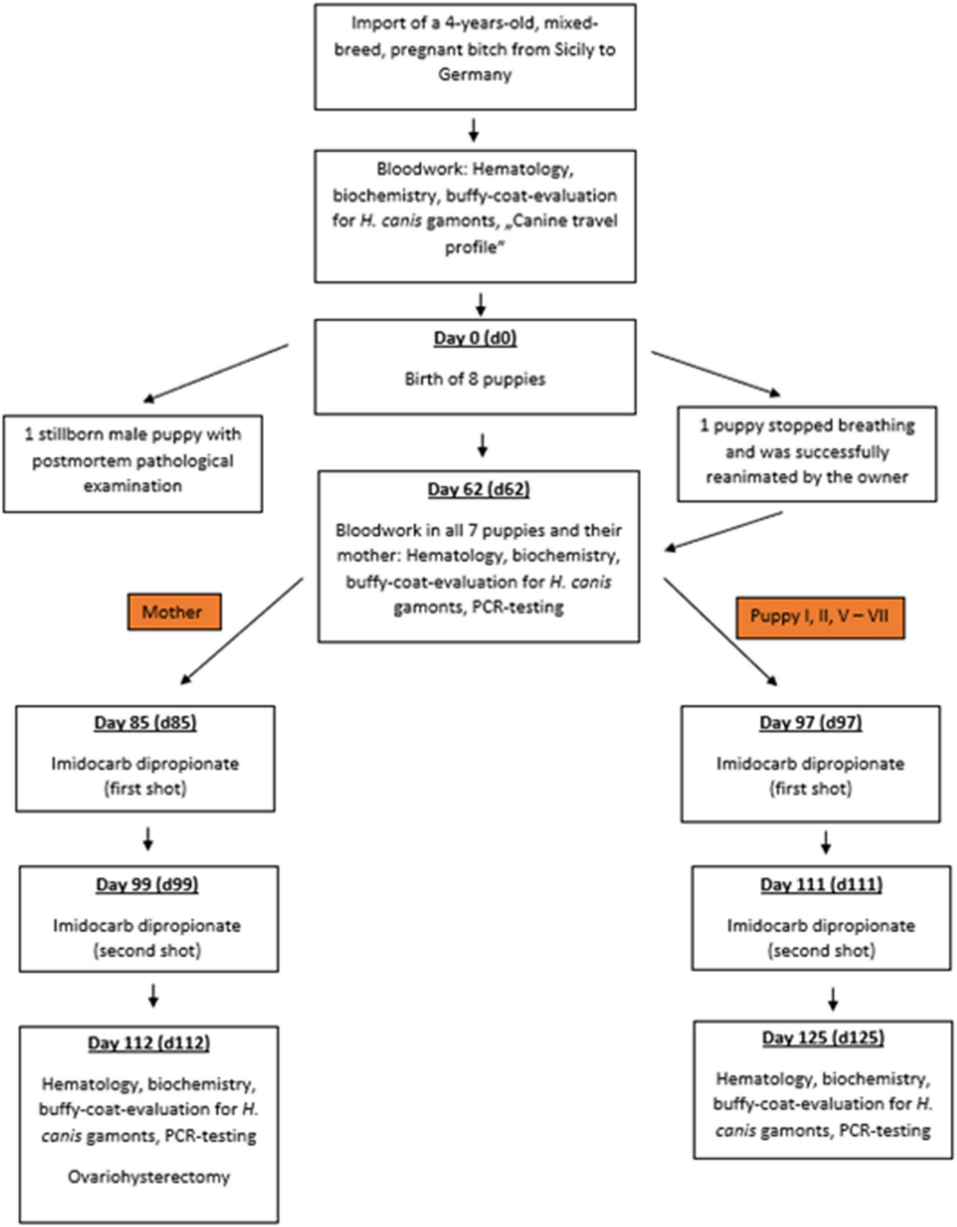
Study design and timeline of a mother dog imported from Sardinia (Italy) to Germany and giving birth to eight puppies, all infected with *Hepatozoon canis*

A 4-year-old, female mixed breed dog was presented to a veterinary practice in Ganderkesee, Germany, with lethargy and tachypnoea in the absence of fever (Fig. 1). The dog was pregnant and was imported from Sardinia (Italy) 2 months prior to the visit. All serological and PCR tests were performed at Laboklin (Bad Kissingen, Germany). The dog tested positive for *Rickettsia* spp. by IFAT (titre 1:512; RICKETTSIA CONORII IFA SLIDE, Viracell, Granada, Spain) and for *H. canis* by PCR (cycle threshold [Ct] 30.3; TaqMan^®^ real-time PCR, in-house test, amplifying a ~ 664-base pair (bp) fragment of the 18S ribosomal [rRNA] gene). Negative test results were found for *Babesia canis* (*Babesia* ELISA Dog, Afosa, Blankenfelde-Mahlow, Germany), *Ehrlichia canis* (*Ehrlichia* ELISA Dog, Afosa, Blankenfelde-Mahlow, Germany), *Leishmania infantum* (Civtest^®^ Canis *Leishmania*, ELISA, Hipra, Amer, Spain), *Anaplasma platys* (TaqMan^®^ real-time PCR, in-house test), *Dirofilaria* spp. (TaqMan^®^ real-time PCR, in-house test for detection of microfilariae) and antigen testing for *Dirofilaria immitis* (FASTest^®^ HW Antigen, MegaCor GmbH, Hörbranz, Austria).

The first haematological examination (day 0; Vet abc Plus+, scil VET) of the mother was performed at the veterinary practice (Table 1) and showed mild anaemia and leucocytosis. All further analyses, including haematology (ADVIA 2120i, Siemens Healthineers) and a biochemistry profile (cobas 2 c 701, Roche Deutschland Holding GmbH) with kidney parameters, liver enzymes and electrolytes, as well as a buffy-coat analysis with quantification of *H. canis*-gamonts were carried out at Laboklin (Table 1). The *Rickettsia conorii* IFAT of the mother was repeated at day 62 and was still positive with a titre of 1:256. The dog also tested positive for *H. canis* by PCR on day 62 (Ct 30.8) and day 112 (Ct 31.8). On day 62, the anaemia had gone, but a mild leucocytosis with mild neutrophilia, lymphocytosis and eosinophilia was present (Table 1). Gamonts were detected in neutrophilic granulocytes on day 0 (4%), day 62 (2%) and day 112 (8%), indicating a moderate *H. canis* concentration in the peripheral blood (Table 1). On day 112, mild lymphocytosis and mild eosinophilia were seen. Biochemistry results were unremarkable apart from a mild initial decrease in iron on day 0 and day 112 as well as a mild hyperkalaemia on day 112 (Additional file 1). The dog was treated twice with imidocarb dipropionate (Carbesia^®^ ad us. Vet., 0.5 ml/10 kg of body weight subcutaneously) on day 85 and day 99. On day 112, the *H. canis* PCR was still positive (Ct 31.8).

**Table 1:** Complete blood count results of a female dog infected with *Hepatozoon canis* at the time of first presentation (day 0), 62 days post-partum and 112 days post-partum

Parameter	Reference intervals^a^	Day 0	Reference intervals^b^	Day 61 post-partum	Day 112 post-partum
RBC	5.5–8.5 × 10^12^/l	5.9	5.5–8.5 × 10^12^/l	7.24	7.06
HGB	150–200 g/l	**133**	150–190 g/l	162	164
HCT	0.44–0.57 l/l	**0.36**	0.44–0.52 l/l	0.45	0.49
MCV	60.0–77.0 fl	61.0	60.0–77.0 fl	63.0	–
MCH	17.0–26.0 pg	22.6	17.0–26.0 pg	22.0	–
MCHC	31.0–38.0 g/dl	37.0	31.0–38.0 g/dl	**36.0**	–
RET	–	–	< 110.0/nl	58.6	**205.4**
CHr	–	–	> 20.1 pg	23.5	27.2
WBC	6.0–12.0 × 10^9^/l	**13.3**	6.0–12.0 × 10^9^/l	**14.4**	10.7
Seg^c^	–	–	3.0–9.0 × 10^9^/l	**9.5**	5.4
Lymph^c^	–	–	1.0–3.6 × 10^9^/l	**3.7**	**3.9**
Mono^c^	–	–	0.04–0.5 × 10^9^/l	0.4	0.5
Eo^c^	–	–	0.04–0.6 × 10^9^/l	**0.7**	**1.0**
Baso^c^	–	–	< 0.04 × 10^9^/l	0.0	0.0
Bands^c^	–	–	< 0.5 × 10^9^/l	0.0	0.0
PLT	200–460 × 10^9^/l	437	150–500 × 10^9^/l	201	157
Hypochr^c^	–	–	Neg	Neg	Neg
Aniso^c^	–	–	Neg	Neg	Neg
*Hepatozoon* gamonts^d^	0%	**4%**	0%	**2%**	**8%**
*Hepatozoon* spp. PCR	Negative	**Positive (ct 30.3)**	–	**Positive** **(ct 30.8)**	**Positive** **(ct 31.8)**

Bold values demonstrate parameters out of the diagnostic thresholds

*RBC* red blood cells; *HGB* haemoglobin; *HCT* haematocrit; *RET* reticulocytes; *CHr* reticulocyte haemoglobin content; *WBC* white blood cells; *Seg* segmented neutrophilic granulocytes; *Lymph* lymphocytes; *Mono* monocytes; *Eo* eosinophilic granulocytes; *Baso* basophilic granulocytes; *Bands* banded neutrophilic granulocytes; *PLT* platelets; *Hypochrom* hypochromasia; *Aniso* anisocytosis

^a^In-house laboratory of the veterinarian (Vet abc Plus+, scil VET, Germany)

^b^Laboklin GmbH & Co. KG. (ADVIA 2120i, Siemens Healthineers, Germany)

^c^Manual differential count (Laboklin GmbH & Co. KG.)

^d^Manual count out of buffy-coat smear (Laboklin GmbH & Co. KG.)

The mother gave birth to eight puppies on day 15 after her first presentation in the veterinary clinic. One of the puppies was stillborn (Fig. 1). A post-mortem examination of the stillborn puppy including histopathology showed that the animal was in a state of advanced autolysis and putrefaction, but the umbilical cord was normal, and no gross malformations were observed. The lungs were not ventilated, and the placenta was not available. As far as recognizable by routine histopathology, the lungs and kidneys showed immature morphology. There was no indication of inflammation in any of the examined organs (lungs, heart, liver, spleen, kidneys, brain). Due to the poor state of preservation, no pathogen-specific abnormalities were recognizable. DNA isolated from the umbilical cord (Ct 31.3), spleen (Ct 35.7) and amniotic fluid (Ct 32.3) were positive for *H. canis* by PCR, whereas the PCRs on the bone marrow and liver were negative.

Six out of the seven surviving puppies were alive at birth, but one dog stopped breathing immediately after birth and had to be reanimated by the owner. This animal (puppy VII) later became ill with fever (40.0–41.0 °C rectal temperature), inappetence and lethargy from day 95 onwards. The puppy presented in a lateral position and was unable to stand or walk on day 97 and received intensive care treatment with application of imidocarb dipropionate (Carbesia^®^ ad us. vet., 0.5 ml/10 kg of body weight subcutaneously). The treatment was successful, and the clinical signs disappeared within 3 days after the first injection. All other four puppies of which the owner still took care were also treated with imidocarb dipropionate out of precaution, twice with a 12–14 day interval (Fig. 1) and have not developed clinical signs since. One puppy was adopted by new owners and therefore lost for further analysis.

**Fig. 2:**
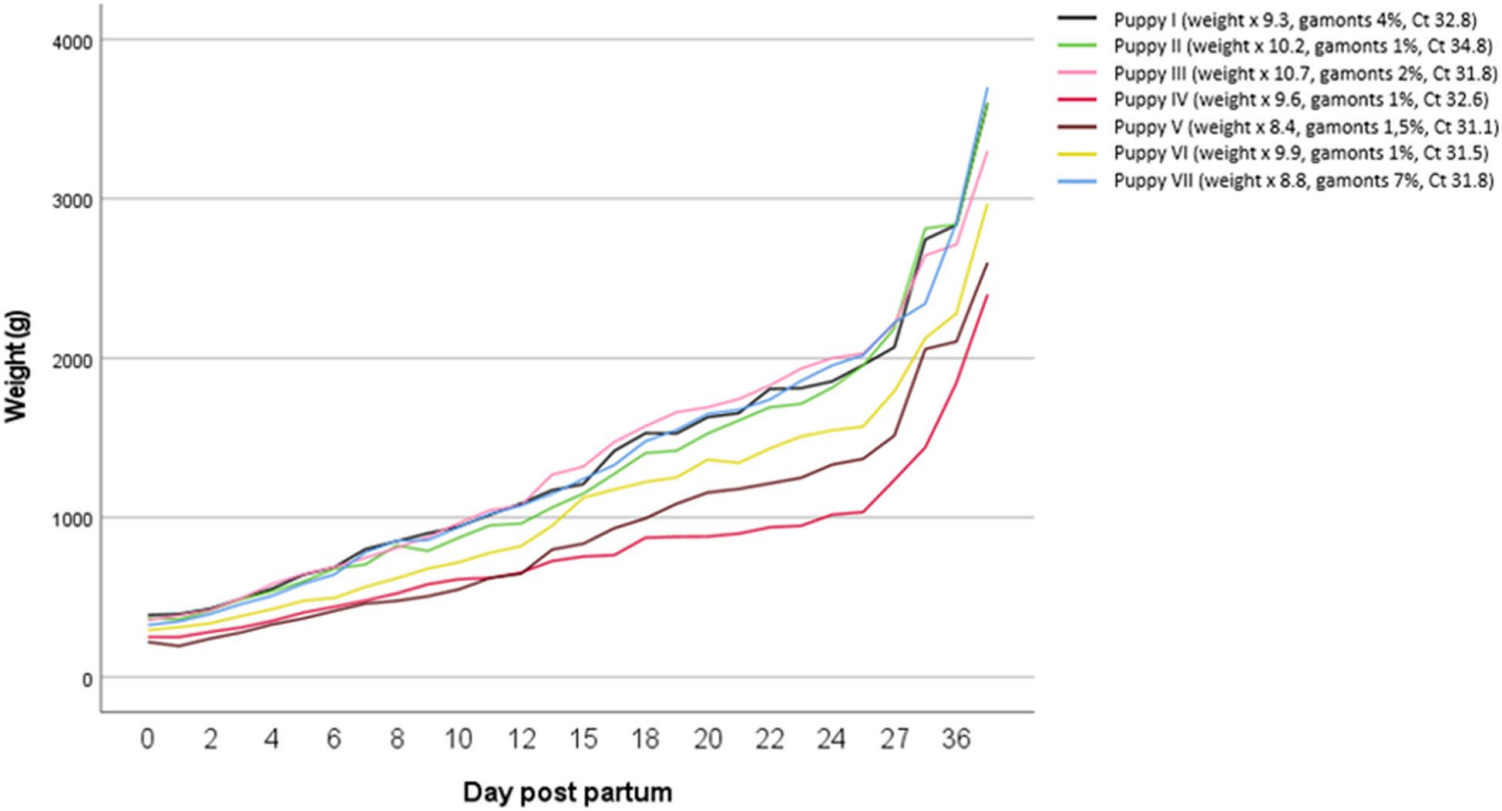
Overview of weight gain in the seven puppies tested positive for *Hepatozoon canis* by PCR from day 0 to day 36 post-partum with amounts of gamonts in buffy coat smears and cycle threshold (Ct) in polymerase chain reaction-testing on day 61 post-partum

In all seven puppies that were alive at the time of writing, haematology was unremarkable, aside from a mild monocytosis in one puppy (Additional file 1). Gamonts of *H. canis* were detected in neutrophilic granulocytes of all puppies, with a range from 1 to 7% (median 1.5%), indicating a moderate concentration of *H. canis* in the peripheral blood. The puppy that had to be reanimated had the highest concentration of gamonts with 7% at day 62 and one of the lowest Ct in PCR testing (31.8; median 31.8, range 31.1–34.8). Biochemistry results revealed mild hyperproteinaemia, a mild increase in albumin and mild hyponatraemia in all puppies. In three out of seven puppies, a mild elevation of urea was seen as well as mild azotaemia in one puppy (Additional file 1: Table S3). At day 125, biochemistry results were available from five out of seven puppies. All five puppies showed a mild increase in creatine kinase and four out of five mild hyperkalaemia. In one puppy, mild elevation in C-reactive protein was recognized as well as decreased urea in another puppy (Additional file 1). The owner monitored the weight of all seven puppies from the day of birth to day 36 (Fig. 2), with the lowest weight gain percentage in dogs with the highest Ct values (puppy V: Ct 31.1, weight gain 8.4%; puppy VI: Ct 31.5, weight gain 9.9%; puppy VII: Ct 31.8, weight gain 8.8%; puppy III: Ct 31.8, weight gain: 10.2%; puppy IV: Ct 32.6, weight gain: 9.6%; puppy I: Ct 32.8, weight gain 9.3%; puppy II: Ct 34.8, weight gain 10.2%).

A ~ 664 base pair fragment of the 18S rRNA gene from *H. canis* was amplified by PCR from samples collected from the bitch, all puppies, and the umbilical cord and amniotic fluid of the stillborn puppy [26]. The PCR products were subsequently sequenced (LGC Genomics, Berlin) and found to be identical to each other. A BLAST analysis of the sequence (GenBank Accession Number ON740944) showed 100% identity to previous *H. canis* entries from Europe, the Americas, and Asia, such as KX712129, MN393911 and MT107098.

To the best of the authors’ knowledge, this is the first study demonstrating the vertical transmission of *H. canis* in a dog in Europe. The diagnosis was made by PCR and detection of gamonts in peripheral blood smears. As the mother dog was imported only shortly before initial screening took place and *R. sanguineus* s.l. ticks are not considered to be endemic in Germany, an infection with *H. canis* of the mother in the country of origin, in this case Sardinia, seems most likely. Due to the absence of the vector and the early detection of the pathogen by PCR after 8 weeks of age (Fig. 1), the infection of the puppies born in Germany most likely occurred vertically. This is supported by the fact that the pathogen was also detected by PCR in the umbilical cord and the spleen of the stillborn puppy, where vector contact would not have been possible. However, an experimental study previously demonstrated that gamonts could be detected in canine blood as early as 28 days post-infection [16]. As the seven surviving puppies in our study were tested for *Hepatozoon* spp. at an age of 62 days for the first time, it cannot be fully excluded that they were infected with *H. canis* after birth. This does seem unlikely, as all puppies were predominantly kept indoors and tick attachment was not observed by the owner.

Screening for co-infections is highly recommended in dogs infected with *H. canis* as clinical signs are mainly observed in animals with co-infections. In our study, we performed a so-called canine travel profile in the bitch. Positive titres in IFAT for *Rickettsia* spp. of 1:512 initially and 1:256 on day 61 were found. As there was no fourfold change in this titre during this period, the results were interpreted as being caused by a past pathogen contact, but a chronic or persistent infection could not be ruled out completely.

Although *H. canis* infections are usually subclinical, some case reports suggest that *H. canis* infections may cause systemic disease in canine puppies, with lethargy, fever, anorexia, weight loss and gastrointestinal signs being reported as the most prominent clinical signs [1, 27, 28]. In our study, immunosuppression due to pregnancy might have been responsible for the reported lethargy and tachypnoea in the mother dog.

The presence and severity of clinical signs is known to correlate with the degree of parasitaemia [22, 29, 30]. The puppy with the highest concentration of *H. canis* gamonts (7%) had to be reanimated, and another puppy with an unknown concentration of gamonts in the peripheral blood was stillborn. Additionally, there might be a correlation between percentage of weight gain and Ct values of the *H. canis* PCR with lowered percentages in dogs with higher concentrations of the pathogen (Fig. 2). However, Ct values are not necessarily proportionate to the level of parasitaemia. A quantitative PCR could not be performed, but quantification of *H. canis* gamonts in the buffy coat. To the best of the authors’ knowledge, it is so far unknown, if the parasitaemia level in puppies may be linked to stillbirth. Further experimental studies may be of interest to clarify this hypothesis.

Mild normochromic anaemia is thought to be the most common clinical abnormality in dogs with *H. canis* infections [1, 31, 32], which was present in the mother dog on day 0 (Table 1). The mild hyperkalaemia on day 112 is most likely linked to mild haemolysis. Lymphocytosis, monocytosis and eosinophilia were recognized in most of the dogs in our study (Tables 1, 2). These haematological findings are in accordance with another study evaluating haematology results in dogs younger than 18 months being infected with *H. canis* [19]. In this study, haematological abnormalities were present in 26 out of 28 dogs (93%), mainly eosinophilia (77%), leucocytosis (46%), lymphocytosis (31%), neutrophilia (23%), monocytosis (19%), thrombocytopenia (19%) and anaemia (4%) [19]. Because 13 out of the 26 dogs (50%) with available information regarding clinical signs and haematological results tested positive for other vector-borne pathogens, only a limited comparison of the mentioned study and our findings is possible. Higher leucocytic count was linked to a higher level of parasitaemia [1], but this was not seen in our study.

**Table 2:** Complete blood count results of 7 puppies infected with Hepatozoon canis at day 62 post-partum performed with ADVIA 2120i [Siemens Healthineers] in the Laboklin laboratory (Bad Kissingen, Germany) with age-related reference intervals according to Rortveit et al. (2015)

Parameter	Reference interval	Puppy I	Puppy II	Puppy III	Puppy IV	Puppy V	Puppy VI	Puppy VII
RBC	4.0–5.5 × 10^12^/l	4.55	4.71	4.58	4.54	4.39	4.47	5.0
HGB	86–177 g/l	98	109	105	103	100	106	111
HCT	0.27–0.37 l/l	0.32	0.36	0.34	0.32	0.31	0.32	0.36
MCV	63.0–74.0 fl	70.0	**77.1**	74.0	70.0	72.0	71.0	71.6
MCH	Pg^a^	22.0	23.1	23.0	23.0	23.0	24.0	22.2
MCHC	29.0–34.0 g/dl	31.0	30.0	31.0	33.0	32.0	33.0	31.0
RET	/nl^a^	177.5	203.5	188.2	205.2	180.9	138.6	187.0
CHr	pg^a^	23.0	24.4	23.6	23.5	22.8	26.3	24.0
WBC	8.8–22.4 × 10^9^/l	13.6	20.5	17.3	15.6	18.9	17.3	17.5
Seg^b^	4.1–12.2 × 10^9^/l	6.8	9.8	6.7	7.2	7.9	8.1	11.9
Lymph^b^	2.7–11.3 × 10^9^/l	4.8	8.6	8.0	7.0	9.1	6.9	3.9
Mono^b^	0.5–1.6 × 10^9^/l	1.4	1.0	**1.7**	0.9	1.5	0.9	0.9
Eo^b^	0.1–1.8 × 10^9^/l	0.7	1.0	0.7	0.5	0.4	1.4	0.9
Baso^b^	× 10^9^/l	0.0	0.0	0.0	0.0	0.0	0.0	0.0
Bands^b^	< 0.5 × 10^9^/l	0.0	0.0	0.0	0.0	0.0	0.0	0.0
PLT	193–653 × 10^9^/l	241	399	367	429	280	393	307
Hypochr^b^	Neg	Neg	Neg	Neg	Neg	Neg	Neg	Neg
Aniso^b^	Neg	Neg	Neg	Neg	**Pos**	Neg	Neg	Neg
*Hepatozoon* gamonts^c^ (day 62) (%)	0	**4**	**1**	**2**	**1**	**1.5**	**1**	**7**
*Hepatozoon* gamonts^c^ (day 125)	0%	**1%**	**1%**	–	–	0%	**1%**	0%
*Hepatozoon* spp. PCR (day 62)	Negative	**Positive (ct 32.8)**	**Positive (ct 34.8)**	**Positive (ct 31.8)**	**Positive (ct 32.6)**	**Positive (ct 31.1)**	**Positive (ct 31.5)**	**Positive (ct 31.8)**
*Hepatozoon* spp. PCR (day 125)	Negative	**Positive (ct 34.3)**	Negative	–	–	**Positive (ct 36.1)**	**Positive (ct 33.1)**	**Positive (ct 33.0)**

Bold values demonstrate parameters out of the diagnostic thresholds

*RBC* red blood cells; *HGB* haemoglobin; *HCT* haematocrit; *RET* reticulocytes; *CHr* reticulocyte haemoglobin content; *WBC* white blood cells; *Seg* segmented neutrophilic granulocytes; *Lymph* lymphocytes; *Mono* monocytes; *Eo* eosinophilic granulocytes; *Baso* basophilic granulocytes; *Bands* banded neutrophilic granulocytes; *PLT* platelets; *Hypochrom* hypochromasia; *Aniso* anisocytosis

^a^No reference values provided by Rortveit et al. (2015)

^b^Manual differential count (LABOKLIN GmbH & Co. KG.)

^c^Manual count out of buffy-coat smear (LABOKLIN GmbH & Co. KG.)

The mild elevation of creatine kinase observed in the five puppies with complete follow-up on day 125 must be interpreted with caution. To the best of the authors’ knowledge, there are no published age-related reference intervals fitting with the age of the puppies. Therefore, an interpretation as age-related changes must be taken into consideration. Additionally, the formation of cysts in the muscular tissue of the puppies due to the *H. canis* infection [33] with subsequent elevation of enzyme activity in the blood can be discussed although no lameness or muscular pain was reported. Interestingly, elevated C-reactive protein was reported on day 125 2 weeks after the second shot of imidocarb dipropionate in the puppy with severe clinical signs, though not in any of the other puppies.

The therapeutic approach for canine *H. canis* infections is challenging, as no drug is officially labelled for treatment of this infection for dogs in Europe. Therefore, treatment options were discussed with the owner and the local veterinary authorities were asked for permission to apply imidocarb dipropionate. Previous reports indicated that treatment with imidocarb dipropionate did not sterilize *H. canis* infections at the standard recommended dose [34]. This was also demonstrated in our study, in which positive PCR results were still observed in the mother and four of the five treated puppies. However, in most of the dogs in our study, the Ct values of PCR tests revealed a lower parasitaemia after treatment. Clinical signs improved quickly in the diseased puppy and the mother was without clinical signs after treatment too. This is concordant with literature as the prognosis of dogs infected with *H. canis* is reportedly good in cases of low parasitaemia, although the decrease in the parasitaemia may be slow and may require several repeated treatments with imidocarb dipropionate [1].

Besides vector-based transmission, vertical transmission of *H. canis* from mother dogs to their puppies may present an important route of transmission. Dog breeders and veterinarians should be aware of this potential risk. As dogs usually do not show clinical signs or clinicopathological abnormalities upon *H. canis* infections, routine screening of dogs imported from endemic countries is important to identify infected animals. It is recommended to perform PCR-testing of both peripheral whole blood and buffy coat to increase the sensitivity. A possible link between stillbirth and *H. canis* infections has to be investigated further, as well as the routes of transmission from bitches to puppies. Our data suggest an impact of the umbilical cord (transmission via blood) while the impact of the amniotic fluid is in doubt. Additionally, further studies are required for evaluation of alternative treatment options in dogs infected with *H. canis.*

## Supplementary Information

**Additional file 1: Table S1**: Biochemistry results of a female dog infected with *Hepatozoon canis* at the time of first presentation (day 0), 62 days post-partum and 112 days post-partum performed with cobas 2 c 701 (Roche Deutschland Holding GmbH) in the laboratory Laboklin (Bad Kissingen, Germany). **Table S2**: Complete blood count results for seven puppies infected with *Hepatozoon canis* at day 125 post-partum performed with ADVIA 2120i [Siemens Healthineers] in the laboratory Laboklin (Bad Kissingen, Germany). **Table S3**: Biochemistry results of 7 puppies infected with *Hepatozoon canis* at day 62 post-partum performed with cobas 2 c 701 (Roche Deutschland Holding GmbH) in the laboratory Laboklin (Bad Kissingen, Germany) with age-related reference intervals according to Rortveit et al. (2015). **Table S4**: Biochemistry results of seven puppies infected with *Hepatozoon canis* at day 125 post-partum performed with cobas 2 c 701 (Roche Deutschland Holding GmbH) in the laboratory Laboklin (Bad Kissingen, Germany).

## Data Availability

All data generated or analysed during this study are included in this published article.

## Abbreviations

Ab-ELISA: Antibody enzyme-linked immunosorbent assay
Ct: Cycle threshold in polymerase chain reaction-testing
DNA: Deoxyribonucleic acid
EDTA: Ethylenediaminetetraacetic acid
IFAT: Immunofluorescence antibody test
PCR: Polymerase chain reaction
