# Supplementary material for: First evidence of vertical Hepatozoon canis transmission in dogs in Europe
Source: Parasit Vectors. 2022 Aug 23;15:296. doi: 10.1186/s13071-022-05392-7 (PMC9396588; doi:10.1186/s13071-022-05392-7)
Supplement: Supplementary file 1 — Additional file 1: Table S1: Biochemistry results of a female dog infected with Hepatozoon canis at the time of first presentation (day 0), 62 days post-partum and 112 days post-partum performed with cobas 2 c 701 (Roche Deutschland Holding GmbH) in the laboratory Laboklin (Bad Kissingen, Germany). Table S2: Complete blood count results for seven puppies infected with Hepatozoon canis at day 125 post-partum performed with ADVIA 2120i [Siemens Healthineers] in the laboratory Laboklin (Bad Kissingen, Germany). Table S3: Biochemistry results of 7 puppies infected with Hepatozoon canis at day 62 post-partum performed with cobas 2 c 701 (Roche Deutschland Holding GmbH) in the laboratory Laboklin (Bad Kissingen, Germany) with age-related reference intervals according to Rortveit et al. (2015). Table S4: Biochemistry results of seven puppies infected with Hepatozoon canis at day 125 post-partum performed with cobas 2 c 701 (Roche Deutschland Holding GmbH) in the laboratory Laboklin (Bad Kissingen, Germany). [file 13071_2022_5392_MOESM1_ESM.docx]

**Table 1:** Biochemistry results of a female dog infected with Hepatozoon canis at the time of first presentation (day 0), 62 days post-partum and 112 days post-partum performed with COBAS 2 C701 (Roche Deutschland Holding GmbH) in the laboratory Laboklin (Bad Kissingen, Germany)

| Parameter | Reference intervals | Day 0 | Day 61 post- partum | Day 112 post- partum |
| --- | --- | --- | --- | --- |
| Alpha-Amylase | <1,650.0 U/l | 636.0 | 791.0 | 631.0 |
| DGGR lipase | <120.0 U/l | 56.2 | 91.7 | 109.9 |
| Glucose | 3.05 – 6.1 mmol/l | 3.1 | 3.3 | 3.7 |
| Fructosamine | <374.0 µmol/l | 243.9 | 253.9 | 316.7 |
| Triglycerides | <3.9 mmol/l | 0.78 | 0.88 | 0.6 |
| Cholesterol | 3.1 – 10.1 mmol/l | 5.0 | 5.2 | 3.8 |
| Bilirubin | <3.4 µmol/l | 0.7 | 1.1 | 1.7 |
| AP | <108.0 U/l | 13.0 | 22.0 | 10.0 |
| GLDH | <6.0 U/l | 1.9 | 2.1 | 1.1 |
| G-GT | <5.0 U/l | 2.6 | 1.2 | 2.4 |
| ALT | <55.0 U/l | 6.7 | 12.8 | 8.7 |
| AST | <25.0 U/l | 12.6 | 13.0 | 7.9 |
| CK | <90.0 U/l | 84.0 | 69.0 | 58.0 |
| Protein | 54.0 – 75.0 g/l | 59.6 | 70.1 | 63.6 |
| Albumin | 25.0 – 44.0 g/l | 37.0 | 37.9 | 40.6 |
| Globulin | <45.0 g/l | 22.6 | 32.2 | 23.0 |
| Urea | 3.3 – 8.3 mmol/l | 5.6 | 6.9 | 3.3 |
| Creatinine | 35.0 – 106.0 µmol/l | 45.0 | 52.0 | 86.0 |
| Phosphorus | 0.7 – 1.6 mmol/l | 1.4 | 1.5 | 1.2 |
| Magnesium | 0.6 – 1.3 mmol/l | 0.8 | 0.8 | 0.8 |
| Calcium | 2.3 – 3.0 mmol/l | 2.4 | 2.7 | 2.5 |
| Sodium | 140.0 – 155.0 mmol/l | 153.0 | 143.0 | 152.0 |
| Potassium | 3.5 – 5.1 mmol/l | 4.8 | 4.5 | **5.2** |
| Iron | 15.0 – 45.0 µmol/l | **14.7** | 20.5 | **14.0** |
| CRP | <15.0 mg/l | - | 1.4 | 1.2 |
| Cortisol | 5.0 – 65.0 ng/ml | 30.6 | 21.3 | - |

AP = alkaline phosphatase; GLDH = glutamate dehydrogenase; G-GT = gamma glutamyl transpeptidase; ALT = alanine aminotransferase; AST = aspartate aminotransferase; CK = creatine kinase; CRP = c-reactive protein

**Table 2**: Complete blood count results of 7 puppies infected with Hepatozoon canis at day 125 post-partum performed with ADVIA 2120i [Siemens Healthineers] in the laboratory Laboklin (Bad Kissingen, Germany)

| Parameter | Reference intervall | Puppy I | Puppy II | Puppy III | Puppy IV | Puppy V | Puppy VI | Puppy VII |
| --- | --- | --- | --- | --- | --- | --- | --- | --- |
| RBC | 5.5 – 8.5 x 10^12^/l | 5.77 | 6.18 | - | - | 5.87 | 6.22 | **5.41** |
| HGB | 150 – 190 g/l | **123** | **142** | - | - | **138** | **147** | **120** |
| HCT | 0.44 – 0.52 l/l | **0.39** | 0.47 | - | - | **0.41** | 0.44 | **0.38** |
| RET | < 110.0/nl | **130.4** | **131.6** | - | - | **181.4** | 103.3 | **129.8** |
| CHr | > 20.1 pg | 26.5 | 28.0 | - | - | 23.9 | 24.2 | 23.4 |
| WBC | 6.0 – 12.0 x 10^9^/l | **13.6** | **14.4** | - | - | **18.1** | **19.1** | **19.4** |
| Seg^A^ | 3.0 – 9.0 x 10^9^/l | 7.5 | 5.2 | - | - | 8.3 | **13.0** | **12.2** |
| Lymph^A^ | 1.0 – 3.6 x 10^9^/l | **3.9** | **7.6** | - | - | **8.3** | **4.6** | **6.0** |
| Mono^A^ | 0.04 – 0.5 x 10^9^/l | **1.6** | 0.4 | - | - | 0.4 | **1.5** | **0.8** |
| Eo^A^ | 0.04 – 0.6 x 10^9^/l | 0.5 | **1.2** | - | - | **1.1** | 0.0 | 0.4 |
| Baso^A^ | < 0.04 x 10^9^/l | 0.0 | 0.0 | - | - | 0.0 | 0.0 | 0.0 |
| Bands^A^ | < 0.5 x 10^9^/l | 0.0 | 0.0 | - | - | 0.0 | 0.0 | 0.0 |
| PLT | 150-500 x 10^9^/l | 212 | 304 | - | - | 225 | 317 | 244 |
| Hypochr^A^ | Neg. | Neg. | Neg. | - | - | Neg. | Neg. | Neg. |
| Aniso^A^ | Neg. | Neg. | Neg. | - | - | Neg. | Neg. | Neg. |
| *Hepatozoon* gamonts^B^ | 0% | **1%** | **1%** | - | - | 0% | **1%** | 0% |
| *Hepatozoon* spp. PCR | negative | **positive (ct 34.3)** | negative | - | - | **positive (ct 36.1)** | **positive (ct 33.1)** | **positive (ct 33.0)** |

RBC = red blood cells; HGB = hemoglobin; HCT = hematocrit; RET = reticulocytes; CHr = reticulocyte hemoglobin content; WBC = white blood cells; Seg = segmented neutrophilic granulocytes; Lmyph = lymphocytes; Mono = monocytes; Eo = eosinophilic granulocytes; Baso = basophilic granulocytes; Bands = banded neutrophilic granulocytes; PLT = platelets; Hypochrom = hypochromasia; Aniso = anisocytosis

^1^No reference values provided by Rortveit es al. (2015)

^A^manual differential count (Laboklin GmbH & Co. KG.)

^B^manual count out of buffy-coat smear (Laboklin GmbH & Co. KG.)

**Table 3:** Biochemistry results of 7 puppies infected with Hepatozoon canis at day 62 post-partum performed with COBAS 2 C701 (Roche Deutschland Holding GmbH) in the laboratory Laboklin (Bad Kissingen, Germany) with age-related reference intervals according to Rortveit et al. (2015)

| Parameter | Reference intervals | Puppy I | Puppy II | Puppy III | Puppy IV | Puppy V | Puppy VI | Puppy VII |
| --- | --- | --- | --- | --- | --- | --- | --- | --- |
| Alpha-Amylase | 305 - 952 U/l | 402.0 | 414.0 | 400.0 | 406.0 | 430.0 | 332.0 | 393.0 |
| Glucose | 4.2 – 8.0 mmol/l | 5.1 | 4.2 | 4.7 | 4.2 | 4.3 | 4.2 | 4.4 |
| Cholesterol | 4.2 – 8.8 mmol/l | 6.1 | 6.6 | 6.3 | 5.7 | 5.7 | 6.7 | 6.4 |
| Bilirubin | µmol/l^1^ | 1.0 | 1.0 | 0.8 | 0.2 | 0.7 | 0.8 | 0.8 |
| AP | 93 - 221 U/l | 96.0 | 135.0 | 147.0 | 113.0 | 107.0 | 124.0 | 134.0 |
| ALT | 19.0 – 32.0 U/l | 13.0 | 15.5 | 14.6 | 12.7 | 13.6 | 12.9 | 14.4 |
| AST | 19.0 – 40.0 U/l | 16.9 | 22.3 | 21.2 | 17.9 | 18.3 | 13.9 | 17.3 |
| CK | 188.0 – 580.0 U/l | 195.0 | 225.0 | 231.0 | 231.0 | 202.0 | 184.0 | 202.0 |
| Protein | 37.0 – 47.0 g/l | **48.5** | **50.6** | **48.1** | **50.9** | **48.5** | **50.0** | **49.4** |
| Albumin | 20.0 – 31.0 g/l | **32.6** | **34.9** | **33.0** | **33.5** | **32.4** | **34.1** | **33.1** |
| Globulin | 16.0 – 22.0 g/l | **15.9** | **15.7** | **15.1** | 17.4 | 16.1 | **15.9** | 16.3 |
| Urea | 2.2 – 5.2 mmol/l | 4.3 | **5.6** | 4.9 | **5.6** | **5.6** | 4.3 | 3.7 |
| Creatinine | 35.0 – 61.0 µmol/l | 49.0 | **62.0** | 49.0 | 58.0 | 57.0 | 47.0 | 44.0 |
| Phosphorus | 2.5 – 3.4 mmol/l | 2.7 | 2.9 | 2.9 | 2.9 | 3.0 | 2.8 | 2.8 |
| Calcium | 2.6 – 3.0 mmol/l | **2.5** | 2.7 | 2.7 | 2.8 | 2.7 | 2.9 | 2.8 |
| Sodium | 140.0 – 148.0 mmol/l | **138.0** | **138.0** | **139.0** | **139.0** | **139.0** | **139.0** | **137.0** |
| Potassium | 4.7 – 7.7 mmol/l | 5.3 | 5.7 | 5.6 | 6.2 | 5.4 | 5.4 | 5.8 |
| CRP | <15.0 mg/l | 1.7 | 2.1 | 2.3 | 1.9 | 2.5 | 2.9 | 1.7 |

AP = alkaline phosphatase; ALT = alanine aminotransferase; AST = aspartate aminotransferase; CK = creatine kinase; CRP = c-reactive protein

^1^No reference values provided by Rortveit es al. (2015)

**Table 4:** Biochemistry results of 7 puppies infected with Hepatozoon canis at day 125 post-partum performed with COBAS 2 C701 (Roche Deutschland Holding GmbH) in the laboratory Laboklin (Bad Kissingen, Germany)

| Parameter | Reference intervals | Puppy I | Puppy II | Puppy III | Puppy IV | Puppy V | Puppy VI | Puppy VII |
| --- | --- | --- | --- | --- | --- | --- | --- | --- |
| Alpha-Amylase | <1,650.0 U/l | 699.0 | 819.0 | - | - | 688.0 | 586.0 | 673 |
| DGGR lipase | <120.0 U/l | 41.0 | 61.9 | - | - | 38.5 | 45.8 | 32.3 |
| Glucose | 3.05 – 6.1 mmol/l | 3.1 | 3.6 | - | - | 3.2 | 3.3 | **2.7** |
| Fructosamine | < 374.0 µmol/l | 302.1 | 251.2 | - | - | 290.3 | 265.7 | 303.9 |
| Triglycerides | < 3.9 mmol/l | 0.71 | 0.64 | - | - | 0.65 | 1.55 | 1.02 |
| Cholesterol | 3.1 – 10.1 mmol/l | 8.2 | 6.7 | - | - | 7.7 | 6.9 | 7.9 |
| Bilirubin | < 3.4 µmol/l | 1.3 | 1.0 | - | - | 1.3 | 1.4 | 0.9 |
| AP | 30.0 – 205.0 U/l^1^ | 70.0 | 117.0 | - | - | 89.0 | 115.0 | 102.0 |
| GLDH | < 6.0 U/l | 2.5 | 2.5 | - | - | 2.3 | 2.7 | 2.4 |
| G-GT | < 5.0 U/l | 2.0 | 1.5 | - | - | 1.2 | 2.0 | 2.3 |
| ALT | < 55.0 U/l | 14.8 | 14.3 | - | - | 11.8 | 17.2 | 16.8 |
| AST | < 25.0 U/l | 20.1 | 14.6 | - | - | 16.4 | 17.5 | 18.9 |
| CK | < 90.0 U/l | **177.0** | **118.0** | - | - | **153.0** | **147.0** | **151.0** |
| Protein | 49.0 – 67.0 g/l^1^ | 51.1 | 52.9 | - | - | 53.6 | 55.3 | 51.7 |
| Albumin | 25.0 – 44.0 g/l | 33.8 | 37.5 | - | - | 37.0 | 37.9 | 33.9 |
| Globulin | < 45.0 g/l | 17.3 | 15.4 | - | - | 16.6 | 17.4 | 17.8 |
| Urea | 3.3 – 8.3 mmol/l | **3.0** | 3.8 | - | - | 7.3 | 5.9 | 3.9 |
| Creatinine | 35.0 – 106.0 µmol/l | 44.0 | 39.0 | - | - | 59.0 | 60.0 | 46.0 |
| Phosphorus | 0.81 – 2.91 mmol/l^1^ | 2.6 | 2.8 | - | - | 2.9 | 2.7 | 2.7 |
| Magnesium | 0.6 – 1.3 mmol/l | 0.8 | 0.8 | - | - | 0.8 | 0.8 | 0.8 |
| Calcium | 1.95 – 3.43 mmol/l^1^ | 2.7 | 2.7 | - | - | 2.6 | 2.8 | 2.8 |
| Sodium | 140.0 – 155.0 mmol/l | 153.0 | 148 | - | - | 148 | 148 | 146 |
| Potassium | 3.5 – 5.1 mmol/l | **5.2** | **5.6** | - | - | **5.7** | 5.0 | **5.2** |
| Iron | 15.0 – 45.0 µmol/l | 23.2 | 30.0 | - | - | 42.6 | 37.4 | 13.3 |
| CRP | <15.0 mg/l | 6.7 | 3.3 | - | - | 3.4 | 3.3 | **25.1** |

AP = alkaline phosphatase; GLDH = glutamate dehydrogenase; G-GT = gamma glutamyl transpeptidase; ALT = alanine aminotransferase; AST = aspartate aminotransferase; CK = creatine kinase; CRP = c-reactive protein

^1^reference intervals according to: Harper et al. (2003)
